# EEG Beta Oscillations in the Temporoparietal Area Related to the Accuracy in Estimating Others' Preference

**DOI:** 10.3389/fnhum.2018.00043

**Published:** 2018-02-09

**Authors:** Jonghyeok Park, Hackjin Kim, Jeong-Woo Sohn, Jong-ryul Choi, Sung-Phil Kim

**Affiliations:** ^1^Department of Human Factors Engineering, Ulsan National Institute of Science and Technology, Ulsan, South Korea; ^2^Department of Psychology, Korea University, Seoul, South Korea; ^3^Medical Device Development Center, Daegu-Gyeongbuk Medical Innovation Foundation, Daegu, South Korea

**Keywords:** thin-slicing, preference, prediction, EEG, beta oscillation, temporoparietal junction

## Abstract

Humans often attempt to predict what others prefer based on a narrow slice of experience, called thin-slicing. According to the theoretical bases for how humans can predict the preference of others, one tends to estimate the other's preference using a perceived difference between the other and self. Previous neuroimaging studies have revealed that the network of dorsal medial prefrontal cortex (dmPFC) and right temporoparietal junction (rTPJ) is related to the ability of predicting others' preference. However, it still remains unknown about the temporal patterns of neural activities for others' preference prediction through thin-slicing. To investigate such temporal aspects of neural activities, we investigated human electroencephalography (EEG) recorded during the task of predicting the preference of others while only a facial picture of others was provided. Twenty participants (all female, average age: 21.86) participated in the study. In each trial of the task, participants were shown a picture of either a target person or self for 3 s, followed by the presentation of a movie poster over which participants predicted the target person's preference as liking or disliking. The time-frequency EEG analysis was employed to analyze temporal changes in the amplitudes of brain oscillations. Participants could predict others' preference for movies with accuracy of 56.89 ± 3.16% and 10 out of 20 participants exhibited prediction accuracy higher than a chance level (95% interval). There was a significant difference in the power of the parietal alpha (10~13 Hz) oscillation 0.6~0.8 s after the onset of poster presentation between the cases when participants predicted others' preference and when they reported self-preference (*p* < 0.05). The power of brain oscillations at any frequency band and time period during the trial did not show a significant correlation with individual prediction accuracy. However, when we measured differences of the power between the trials of predicting other's preference and reporting self-preference, the right temporal beta oscillations 1.6~1.8 s after the onset of facial picture presentation exhibited a significant correlation with individual accuracy. Our results suggest that right temporoparietal beta oscillations may be correlated with one's ability to predict what others prefer with minimal information.

## Introduction

Humans have an ability to find information based on a narrow slice of experience, called “thin-slicing” (Gladwell, 2007). Thin-slicing is often used for judgments about other people, which are fairly accurate even only from a brief observation (Albright et al., 1988; Funder and Colvin, 1988; Watson, 1989). Furthermore, limited slices of experience such as a mute video, a facial appearance picture, or a gait pattern have been shown to subserve accurate prediction about the altruism, sex orientation, violence, or reliability of others (Engell et al., 2007; Johnson et al., 2007; Van't Wout and Sanfey, 2008; Fetchenhauer et al., 2010; Stillman et al., 2010). A few putative theories have attempted to explain how thin-slicing works. The first theory, taking an ecological approach, suggests that humans can quickly recognize key features of others (e.g., angry or fear faces) using thin-slices of experience to promote survival and adaptation (McArthur and Baron, 1983). The second theory based on common stereotypes and social expectations suggests that humans make an initial judgment based on the memory of common stereotypes as well as social behavioral conformation to expectations of others (Snyder et al., 1977; Banaji et al., 1993). The third theory, focusing on stimulus information processing, suggests that thin-slicing works by minimizing excessive thinking of self-presentation while enhancing the capability of dealing with others' information (Gilbert and Krull, 1988). So far, however, no single theory stands out as a comprehensive explanation of the mechanism of thin-slicing.

A number of studies have uncovered the characteristics of thin-slicing. Hall described that female is better at decoding nonverbal communications than male presumably due to traditional social positions of female (Hall, 1978). Ambady and Rosenthal characterized several aspects of the judgment about others through thin-slicing as follows (Ambady and Rosenthal, 1992): First, the judgment accuracy through thin-slicing is independent of the observation length of a stimulus as long as the observation is made within 5 min. Albrechtsen et al. supported these characteristics of thin-slicing by showing that exposure to 15-s video stimuli led to better discrimination rates than exposure to 3-min video stimuli in social judgment tasks (Albrechtsen et al., 2009). Second, the judgment accuracy does not depend on a type of stimuli; whether it is verbal or non-verbal. Third, seeing both face and body leads to a more accurate judgment than seeing only one of them but additional hearing of speech does not increase accuracy further. These observations suggest that judgments through thin-slicing may not need a prolonged exposure to stimuli or excessive sensory information in order to increase accuracy.

Among many types of judgments made possible by thin-slicing, the present study focuses on the prediction of the preference of others through thin-slicing (North et al., 2010; Kang et al., 2013) due to a few experimental advantages of preference prediction. First, preference can be readily predicted as a binary choice such as liking vs. disliking. Also, judgments of others' preference over particular items may allow us to dissect cognitive processes during thin-slicing, such as the acquisition of others' information and the prediction of their preference over a given item. One can separate these processes by first providing the information of others to a person followed by showing an item and asking the person to predict whether the person would prefer it or not. This temporal segregation of preference prediction processes is particularly useful for our study aim at understanding temporal brain activity patterns during thin-slicing. A theoretical basis, called the projection theory, for preference prediction through thin-slicing has proposed that when a person predicts the preference of others, the person tends to consider their own liking and estimate the other's preference according to a perceived difference between the other and self (Hoch, 1987, 1988; West, 1996). Other studies have also shown that similarities between self and others as well as self-opinions can affect prediction results (Kurt and Inman, 2013). Therefore, differential processing of the information of self and others may underlie the prediction of what others prefer through thin-slicing.

A number of neurophysiological studies have investigated brain activities related to the information processing of self and others (Frith and Frith, 1999, 2003; Vogeley et al., 2001; Kampe et al., 2003; Platek et al., 2004; Seger et al., 2004; Johnson et al., 2005; Jimura et al., 2010; Jung et al., 2013; Cook, 2014). Using functional resonance imaging (fMRI), the previous studies found neural activations over a number of brain regions, including medial prefrontal cortex (mPFC), anterior cingulate cortex (ACC), and superior parietal lobule, when people took either the perspective of others or self (Frith and Frith, 1999; Seger et al., 2004; Johnson et al., 2005; Jung et al., 2013). In particular, dorsomedial prefrontal cortex (dmPFC), temporal pole, bilateral temporoparietal junction (TPJ), posterior cingulate cortex (PCC), and posterior superior temporal sulcus (STS) were activated when people took the perspective of others (Frith and Frith, 1999, 2003; Vogeley et al., 2001; Kampe et al., 2003; Platek et al., 2004; Jung et al., 2013). On the other hand, rostomedial prefrontal cortex and pregenual anterior cingulate cortex were activated when people took the perspective of self (Northoff et al., 2006; Denny et al., 2012). A study by Kang et al. reported that mPFC activity was related to information processing of self and others whereas functional connectivity between mPFC and right TPJ or PCC was related to thin-slicing accuracy (Kang et al., 2013).

In spite of mounting evidence about neural substrates of thin-slicing, little is known about the temporal dynamics of neural activity emerging during thin-slicing. Since prediction preference through thin-slicing is likely to involve a number of cognitive processes to address a relatively challenging problem, the temporal dynamics of neural activity during thin-slicing may reveal how these processes interplay to support prediction. In particular, the temporal analysis can help us to find neural substrates of individual abilities to predict what others prefer through thin-slicing, as not everyone has the same level of predictive performance (Kang et al., 2013). In this study, we aim to investigate temporal aspects of brain activity (e.g., when a particular brain activity occurs during thin-slicing) that correlate with individual performance of others' preference prediction through thin-slicing. Taking the aforementioned advantages of preference prediction, we intend to facilitate the temporal analysis by explicitly segregating the thin-slicing task period into two phases: the first phase of the brief acquisition of the information of a target person; and the second phase of predicting whether or not the target person prefers a given item. In addition, to differentiate neural activities related to the processing of the preference of self vs. others, which would be essential to understand preference prediction based on the false consensus effect, we separate the thin-slicing task trials for reporting self-preference from those for predicting others' preference while keeping the same task procedure as well as the same items between them. Since female is reportedly better than male at the judgment of others through thin-slicing (Hall, 1978), we opt to study the behavioral and neural responses of female subjects (Kang et al., 2013).

As to brain activity measurements, we use scalp electroencephalography (EEG) as it provides a higher temporal resolution than fMRI and thus is more suitable to examine time-varying brain patterns over a short period of thin-slicing. Previous studies revealed that social skills such as recognizing others' facial emotions are accompanied by increases in the magnitude of alpha oscillations in EEG (Popova et al., 2014; Kang et al., 2015). EEG beta oscillations have also been used for predicting one's preference on movie trailers or musical tempo (Bauer et al., 2015; Boksem and Smidts, 2015). Beta oscillations indicate experience of pleasure, reward processing and functional coupling of different brain regions. Therefore, we hypothesize that changes of EEG alpha and beta oscillations in time can probe the temporal sequence of neural activity related to prediction of others' preference during thin-slicing. To address this first hypothesis, we analyze EEG oscillations at a series of time windows during the thin-slicing period. Second, we compare EEG oscillations when a person predicts others' preference and when the person reports self-preference to find temporal patterns of EEG oscillations signifying the process of predicting others' preference. Based on one of the thin-slicing theories focusing on stimulus information processing (Gilbert and Krull, 1988), one's ability of predicting what others prefer through thin-slicing may be associated with how well one suppresses thinking about self-preference and at the same time takes the perspective of others by exploring the given information of others. Hence, we hypothesize that differences in neural activities related to self-report of own preference and prediction of others' preference would be correlated with one's ability to predict others' preference through thin-slicing. To address the second hypothesis, we investigate how differences in EEG oscillations at various time windows between the trials of self and others' preference correlate with individual prediction accuracy.

## Materials and methods

### Participants

Twenty right-handed female undergraduate students participated in the study (average age: 21.86, range: 20–25). All participants had no medical history of neurological illness or damages and did not take any psychiatric medicine. All participants were able to keep still their bodies for a long time and fully recognize the images of prediction items. Each participant received a monetary reward of 20,000 KRW after the study. Experimental procedures were approved by the ethical committee of Ulsan National Institute of Science and Technology (UNISTIRB-15-04-C) and the study was conducted in accordance with the Declaration of Helsinki. All research participants were informed of the study aims and experimental procedures. A written consent form was provided by every participant.

### Stimuli

Visual images were prepared to show the facial image of the target persons as well as the image of the items to be predicted (or reported) for preference. We used the same stimulus set as in the previous study (Kang et al., 2013). The overall procedure of selecting the stimulus set was as follows. First, for the selection of the items, we initially collected 280 pictures of random items in five categories of food, movie, bag, shoe, and book (Kang et al., 2013). In particular, for the movie category, the pictures contained publically available movie posters. Then, a separate group of 18 participants was recruited to evaluate their preference over each item with a 4-point scale from 1 (strongly dislike) to 4 (strongly like). From these evaluation outcomes, 10 samples that showed medium levels of preference as well as high variance across participants were selected from each category.

Next, to select the target persons, another separate group of 56 participants (27 males, mean age: 22.78 years, std: 1.95 years) were recruited and reported their preference over 50 selected items (10 from each of 5 categories) with the same 4-point scale as above. The photograph of every participant was also taken, capturing the face with slight smile and the shoulder in front of a gray background. Among 56 participants, we selected nine target persons with a criterion of a high between-subject variability of facial appearance and within-subject variability of preference over items.

**Figure 1:**
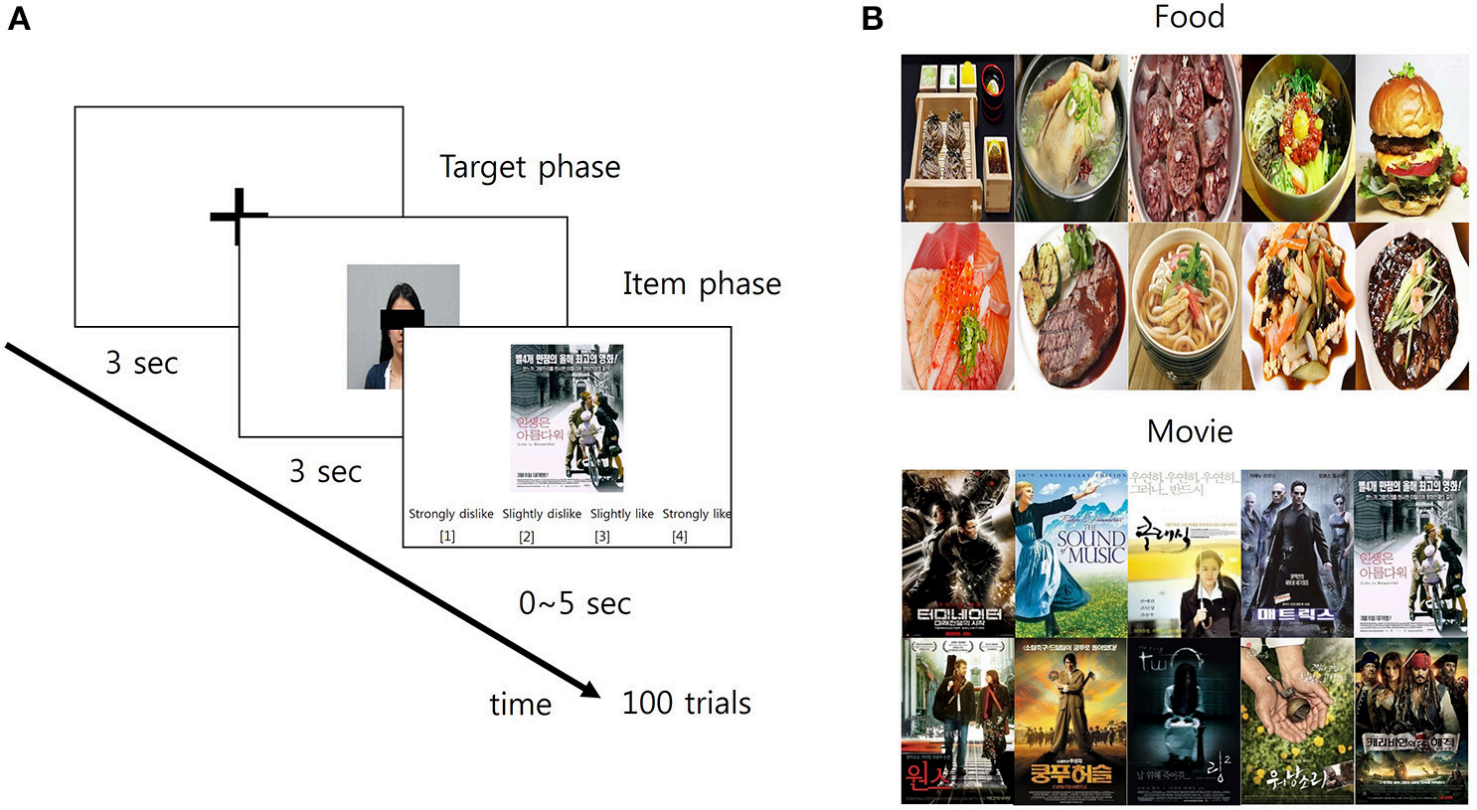
The schematic diagram of the preference prediction task. **(A)** Each trial began with the 3-s fixation period. Then, the facial picture of a target person whose preference was predicted later appeared on the screen for 3 s (target phase). Following the first phase, the picture of an item over which the preference of the target person was predicted appeared on the screen for 5 s (item phase). Within this 5-s period, participants predicted the target's preference over the item using a 4-scale response: (1) strongly dislike; (2) slightly dislike; (3) slightly like; and (4) strongly like. **(B)** The pictures of 10 items in each category (food and movie, respectively) were used in the study.

Together with the selected set of the pictures of the items, the photographs of the target persons and their preference scores over the items, we also took the photographs of the participants in the present study and used them for the self-report of preference. All the visual images were adjusted into an identical frame with the size of 720 × 480 pixels (visual angle: 23.13°). Finally, among five preference categories, we only used the stimuli from the food and movie categories in the present study since only these two categories showed significant preference prediction accuracy in the previous study (Kang et al., 2013; see Figure 1B).

### Experiment procedure

After arriving at the laboratory, participants received a detailed verbal instruction about the procedure and purpose of the experiment. Participants sat on a chair comfortably and took a rest during the EEG setup for ~30 min. Then, they practiced several pretest trials of the preference prediction task before the main experiment in order to minimize errors. The main experiment consisted of two sessions: each for the food and movie category. The order of the sessions was counterbalanced across the participants. Each session contained 100 trials with no break and feedback. A trial began with a baseline period in which a black fixation cross appeared on the center of a white screen for 3 s. The size of the fixation cross was 30 × 30 pixels (visual angle: 1.528°). Then, the preference prediction task period followed. The task period was composed of two successive phases. In the first phase, participants were shown the photograph of one of the target persons or self for 3 s, which was located at the center of the screen. The presentation order of the 10 photographs (9 target persons + 1 self) was randomized. Immediately after the first phase, the second phase followed in which participants were shown the picture of an item. The item picture appeared at the center of the screen until the end of the second phase. The second phase ended either when participants' response was detected or when 5 s elapsed from the onset of the second phase. Participants provided their prediction response by pressing the designated keyboard buttons. The trial was deemed to be failed when participants could not provide their prediction response within 5 s. Participants predicted the preference of the target person for the item by choosing one of four responses which was written by Korean: (1) strongly dislike, (2) slightly dislike, (3) slightly like, and (4) strongly like (corresponding keyboard buttons were D, F, J, and K, respectively). For instance, pressing the “strongly like” button (keyboard button “K”) recorded participants' prediction that the target person would like the item very much. When the self-photograph was shown in the first phase, participants simply reported their preference on a given item with the same response options. Every possible pair of the target person and the item was presented to participants in a random order. Consequently, a total number of trials was 200 (10 targets including self × 20 items). The average time taken for a single trial was 7.5628 ± 0.2242 s. The next trial started with the baseline period immediately after the second phase ended. Hereafter, the trial with the prediction of others' preference is called the “other-trial” and the trial with the report of self-preference is called the “self-trial.” Also, the first phase is called the “target phase” and the second is called the “item phase,” respectively (Figure 1A).

### EEG recordings

The EEG signals of each participant were acquired during the entire experimental period using multiple Ag-AgCl referential active electrodes placed on the scalp and amplified by a commercial EEG amplifier (BrainVision actiChamp, Brain Products GmbH, Gilching, Germany). The sampling rate was 500 Hz. A total of 19 electrodes were placed on the surface of the scalp following the international 10–20 system. The electrode positions, identified by the EEG cap from the vendor (actiCap, Brain Products GmbH, Gilching, Germany), were FP1, FP2, F7, F3, Fz, F4, F8, T3, C3, Cz, C4, T4, T5, P3, Pz, P4, T6, O1, and O2. The ground electrode was placed at the position of FPz. A reference electrode was placed at the right ear. A conductance gel (SuperVisc 1,000 g, Brain Products GmbH, Gilching, Germany) was inserted between each electrode and the surface of the scalp. The impedance was maintained below 10 kΩ throughout the recordings.

### Behavioral data analysis

We calculated the prediction accuracy of each participant by comparing pre-recorded true preference responses of the target persons and predicted responses by the participant. To simplify calculation, we rearranged the four types of responses of the target persons and participants into binary responses as either “like,” including both “strongly like” and “slightly like,” or “dislike,” including both “strongly dislike” and “slightly dislike,” Then, we matched these simplified responses between the target persons and each participant for every item. We excluded those trials where no response of participants was obtained before the 5-s time limit. Consequently, 99.4 ± 1.3917 responses on average for food and 99.9 ± 0.3078 responses on average for movie were included in the analysis of accuracy. Also, we measured response time (RT) for each trial and calculated the average RT for the other-trial and the self-trial, respectively, for each participant. Then, we statistically evaluated a difference in RT between the other-trial and the self-trial using the paired *t*-test.

We estimated a chance level of prediction accuracy for each participant and for each item category, as we suspected that a chance level generated by a random guess depended on a possible bias of the target person's responses in a particular category. For each participant and each category, we first shuffled the order of the original preference prediction results, generating a set of pseudo-random prediction data. Then, we compared the shuffled prediction data with the target persons' true preference data for each item within a given category and calculated prediction accuracy. We repeated this procedure 1,000 times to create a cumulative distribution of random prediction accuracy. Then, we fitted a curve to this cumulative distribution of random accuracy values. Using the fitted cumulative distribution curve, we calculated the 95% percentile and determined it as a chance level of prediction for the given category of the participant. This estimation of the chance level was conducted for every pair of participant and category. We deemed that if one's prediction accuracy was higher than the estimated chance level, the accuracy was significant with 95% confidence.

### Time-frequency analysis of EEG

We filtered the raw EEG signals using a Butterworth filter (4-order zero-phase IIR filter) with a pass band from 0.1 to 50 Hz. Then, we determined an epoch of the EEG data analysis corresponding to each trial, containing EEG signals 1 s before the onset of the target phase to 3 s after the onset of the item phase; a single epoch spanned 7 s, consisting of 1 s of fixation, 3 s of the target phase and 3 s of the item phase. Also, we eliminated the noisy channels (FP1, FP2) and applied the standardized REST methods to avoid a potential issue of lateralized referencing (Yao, 2001; Yao et al., 2005; Dong et al., 2017). Only 96.06% trials were used after outlier elimination (<120 μV).

To analyze the spectral features of EEG signals of each of the 19 EEG channels, the short-time Fourier transform (STFT) was used with a 0.5-s hamming window and a 0.4-s overlap. The frequency resolution was 1.9531 Hz. With these settings, STFT yielded 66 windows × 129 frequency components in each epoch. The Welch's power spectral density estimation of each frequency component at each time window was obtained per epoch. Then, we averaged the power spectral density estimates over all the epochs belonging to each type of trial (other-trial or self-trial). In this experiment, we assumed that the result of EEG analysis would show a continuous interval of more than 0.2 s. So, we processed the moving average below the frequency of 30 Hz. This yielded a 17 × 64 × 15 time-frequency data matrix (i.e. 17 channels, 64 time windows and 15 frequency components) for each of two conditions in each participant. Since the number of self-trial was much smaller than the number of other-trial and the type of stimuli would not influence stimulus information processing much in the self-trial compared to the other-trial, we averaged time-frequency data of the self-trial epochs for both food and movie stimuli whereas we obtained average time-frequency matrices separately for each of food and movie stimuli in the other-trial.

### Statistical analysis

To address the first hypothesis that temporal patterns of EEG oscillations could reveal the temporal aspect of neural activity for the prediction of others' preference, we statistically analyzed within-subject differences in EEG spectral power between the two conditions (self-preference report vs. others' preference prediction) using the paired *t*-test across different time windows. This within-subject analysis examined whether some brain oscillations revealed different patterns when processing others' information at a specific time-frequency combination. To address the second hypothesis that differences in neural activities between reporting self-preference and predicting others' preference would be correlated with individual ability to predict others' preference, we analyzed between-subject correlations between EEG spectral power at a specific time-frequency combination and prediction accuracy using the Pearson correlation analysis. Since these analyses were performed for each combination of time-frequency independently, all the *p*-values were corrected by Bonferroni correction for multiple comparison.

## Results

### Behavioral prediction accuracy

**Figure 2:**
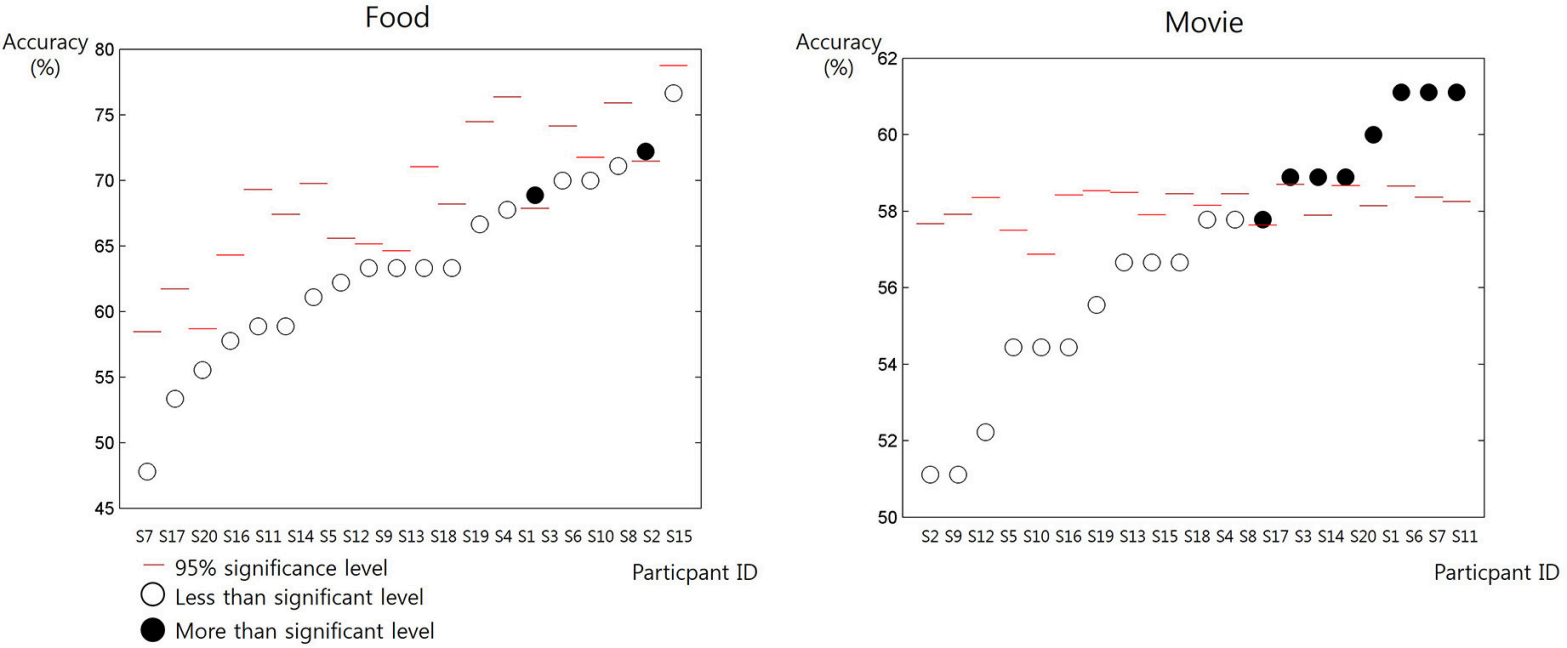
The accuracy of the prediction of others' preference in individual participants (from S1 to S20) for the items in the two categories: food and movie. The red bars denote the chance level of prediction accuracy estimated from the behavior data of individual participants. The white circles denote the resulting prediction accuracy of individual participants lower than the chance level. The black circles denote the resulting prediction accuracy of individual participants higher than the chance level.

The mean and the standard deviation of RT was 1,572 ± 2.775 ms for the self-trial and 1,586 ± 2.274 ms for the other-trial, respectively. The paired *t*-test revealed no significant difference in RT (*p* = 0.7270). When predicting others' preference of movie, 8 out of 20 participants showed significantly prediction accuracy higher than the chance level (permutation test, 95% confidence) (Figure 2). On the contrary, when predicting others' preference of food, only 2 participants showed significantly prediction accuracy higher than the chance level (permutation test, 95% confidence).

### Time-frequency patterns of EEG

**Figure 3:**
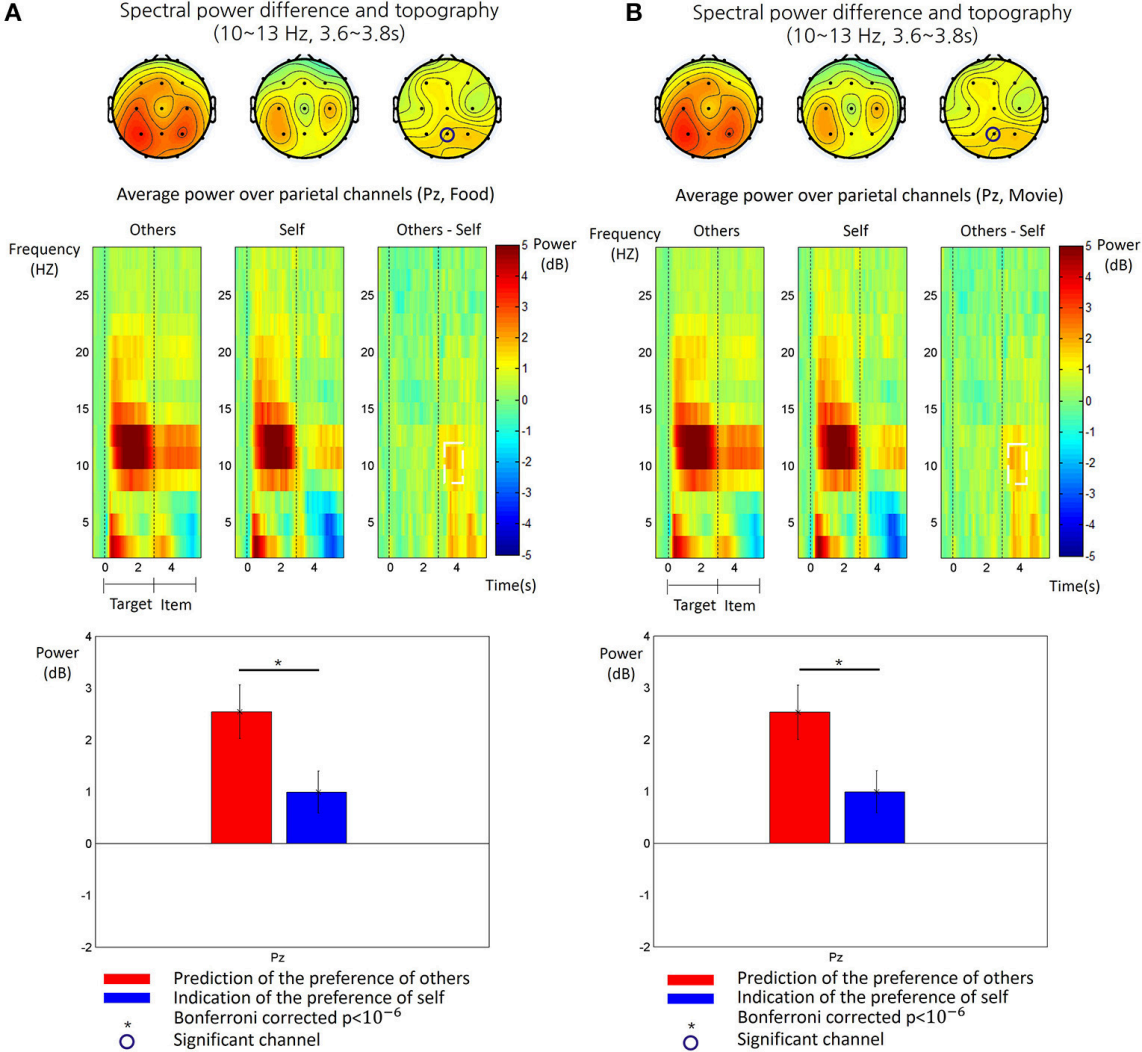
The time-frequency patterns of EEG in food stimulus trials **(A)** and the movie stimulus trials **(B)**, respectively. Top: The topography of the alpha power (10~13 Hz) measured in the period of 3.6~3.8 s after the target phase, for the other-trial (left), the self-trial (center) and differences between them (right). The circled channels (Pz) showed significant differences of alpha power between the other-trial and self-trial. Middle: The time-frequency map of grand average spectral power of EEG recorded at Pz are shown for the epoch from 0.5 s before the target phase to 3 s after the item phase. The time 0 s indicates the onset of the target phase. The spectral power values were baseline-corrected by subtracting the mean value in the baseline period (−0.5~0 s) from all the values, for each frequency. The map shows the time-frequency patterns for the other-trial (left), self-trial (center) and differences between them (other-self, right). The white dotted line box indicated a time-frequency window where a significant difference between the other-trial and self-trial was found (paired *t*-test, *p* < 0.05, Bonferroni corrected). Bottom: The comparison of alpha power between the other-trial and self-trial at Pz. Alpha power in the other-trial was larger than that in the self-trial (paired *t*-test, *p* < 0.05, Bonferroni corrected). The error bar indicates the standard error of the mean.

We obtained the time-frequency patterns of EEG in each participant for the other-trial and the self-trial, respectively. Figure 3 shows the average time-frequency patterns of EEG for each type of trials and subsequent analysis results. In both types, there was a clear increase at the alpha frequency band (10~13 Hz) over the parietal region during the target phase. During the item phase, however, alpha power increase was more consistently pronounced in the other-trial than in the self-trial over the middle parietal regions. Therefore, we analyzed the difference of alpha power between the other-trial and self-trial in the item phase and found a significant difference during a time period of 0.6~0.8 s after onset of the item phase at Pz (paired *t*-test, *p* < 0.05, Bonferroni correction). This significant difference was also shown for the both food and movie stimuli (see Figure 3).

### Correlation between prediction accuracy and EEG features

**Figure 4:**
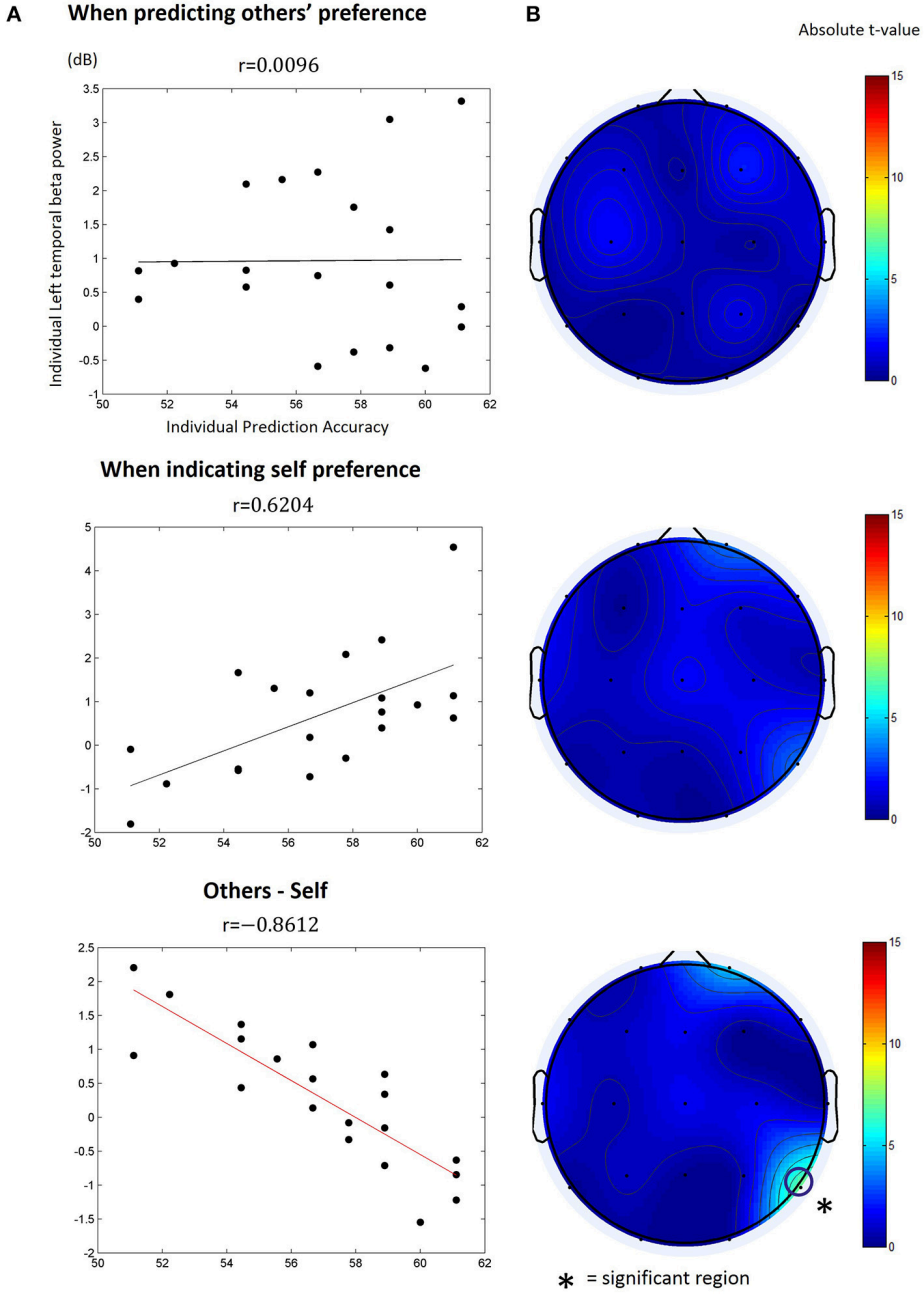
The correlation between individual others' preference prediction accuracy and the beta (13~18 Hz) power at T6 within 1.65~1.85 s after target appearance. **(A)** The beta power value was obtained from (top) the other-trial data where participants predicted other's preference, (middle) the self-trial data where participants reported their preference, or (bottom) differences in the beta power between the other-trial and the self-trial. The dots in the plot indicate individual data points. *r* is the Pearson correlation coefficient. The solid lines denote the estimated a linear relationship between the beta power and prediction accuracy (black: non-significant; red: significant, *p* < 0.05, Bonferroni corrected). **(B)** The topograph of the absolute *t*-value of the estimated correlations. The open circle in the bottom indicates the EEG channel that showed a significant linear correlation with individual prediction accuracy.

We calculated the Pearson correlation between participants' individual prediction accuracy and spectral power of every combination of the frequency band and time window for each type of the trials. However, we found no particular time-frequency combination yielding a significantly high correlation (Bonferroni correction, *p* > 0.05) in both the other-trial and the self-trial data. Next, we calculated the Pearson correlation between participants' individual prediction accuracy and differences of the spectral power between the other-trial and the self-trial over every combination of frequency band and time window. We only found one particular time-frequency combination yielding significant correlations in the movie stimuli (but not in the food stimuli); beta power (13~18 Hz) at channel T6 (right temporoparietal) within the period of 1.65~1.85 s after target appearance was significantly correlated with individual prediction accuracy (*r* = −0.8612, Bonferroni corrected, *p* < 0.05; Figure 4). In other words, individuals with higher prediction accuracy revealed lower levels of beta power at the right temporoparietal area.

## Discussion

In this study, we investigated brain activity related to the prediction of the preference of other persons through thin-slicing. First, we hypothesized that temporal patterns of EEG oscillations would represent the temporal sequence of brain activity related to the prediction of others' preference. Our results showed that alpha power increase was manifested for both the self-trial and other-trial in 0.5~2.5 s after the onset of the target person's face image presentation. But then, entering into the second phase of linking the target person and an item, alpha power increase was pronounced only in the other-trial over parietal region, being absent in the self-trial. Second, we hypothesized that differences in EEG oscillations between self-information processing and others' information processing would be related to one's ability to predict others' preference. Our correlation analysis results showed that there was no significant correlation between EEG spectral power and prediction accuracy for either the other-trial or self-trial. However, we found that the *difference* in the beta power between the other-trial and self-trial, measured at the right temporoparietal region at 1.6~1.8 s after the target image appeared, was significantly correlated with individual prediction accuracy. It shows that differential right temporoparietal beta oscillations observed at the time of association of others' face with an item may be indicative of personal ability to predict others' preference through thin-slicing with facial images. As a result, participants who showed larger differences in right temporoparietal beta power of the self-trial from the other-trial (i.e., other-trial < self-trial) predicted others' preference better.

The result of this study showed no difference in response time between the self-trial and the other-trial. It is opposite to the previous report demonstrating faster response time in the self-trial than in the other-trial (Seger et al., 2004). We suspect that the difference might be due to a difference in the experiment design. In the previous research, the blocks of the self-trial and the other-trial were separated. However, in the present study, both the self-trial and other-trial were randomly mixed within the same block. Hence, in the experimental procedure of the previous research, participants might be able to focus more on the item itself to make a quicker decision in the block of the self-trial, whereas participants in our study might need to persistently retain and integrate the target person's information during the item phase when the self and others randomly appeared in a sequence. This supposition could have been verified by analyzing response times of the trials when the previous target person appeared again, but we failed to do it due to the lack of a sufficient number of such trials in our data. Nevertheless, our result of response time indicates that dependency of EEG activity on the target (i.e., self vs. others) might be irrelevant of difference in response time.

Acquiring facial information is one of the most common social activities since the face can provide much information such as emotion and personality traits in social communication (Berry and McArthur, 1985; Willis and Todorov, 2006; Jack and Schyns, 2015). The alpha ERS has been shown to occur during the perception of facial expression, which is less pronounced in patients with schizophrenia (Popova et al., 2014). In addition, previous studies of a joint attention task showed a difference in alpha oscillations over the left centro-parieto-occipital area between the cases with and without joint attention (Lachat et al., 2012). Other studies reported that alpha and beta oscillations were modulated in rTPJ when social bargaining activities were performed in normal subjects as well as patient with dementia, Alzheimer's and frontal area loss. The studies also demonstrated that fronto-temporo-parietal network connectivity was associated with self-others integration strategy (Melloni et al., 2016). Alpha activity in rTPJ has been shown to be a predictor of behavioral choices in the Ultimatum game, which was not the case when subjects with schizophrenia played the game or when normal subjects played the game against the computer (Billeke et al., 2015). These previous results indicate that parietal alpha ERS reflects social information processing when humans receive facial information. We also observed alpha ERS when participants acquired facial information of others or self, which was necessary for the subsequent preference prediction. This alpha ERS observed during the view of the faces of both self and others in the target phase might point to the acquisition process of facial information regardless of perspective.

However, the later alpha ERS at the parietal region observed in the item phase showed different patterns between the other-trial and self-trial. Alpha ERS was clearly pronounced only in the other-trials. In the other-trial, participants dealt with social information by linking the presented item with previously acquired facial information of others, while they simply reported their preference on the presented item without any social information processing in the self-trial. Hence, it can be argued that this alpha ERS in the other-trial might still reflect facial information processing as in the target phase since participants needed to retain the facial information of others to predict preference. However, the brain regions exhibiting alpha ERS were slightly different between the target phase (overall posterior regions) and the item phase (middle parietal regions), implying that the second alpha ERS might represent more than just facial processing. Since social information processes in these phases would be different, there is a possibility that alpha ERS shown in the item phase may reflect a participant's social cognitive processing of predicting whether the target person would prefer the presented item. Note that, however, this social cognitive processing would not be correlated with prediction accuracy, because similar alpha ERS was also observed for the food stimuli trials.

We did not find a significant correlation between the spectral power of EEG and individual prediction accuracy for either the other-trial or the self-trial. In contrast, discrepancy of the spectral power of EEG between the other-trial and the self-trial was significantly correlated with individual prediction accuracy, for beta oscillations at the right temporoparietal region. This result may be linked with a manner of information processing in which participants performed social prediction. The previous study reported that individual performance of predicting personal traits of others could be influenced by internal psychological processes; people tended to predict about others by anchoring built from the integration of self-states, stereotypes and experiences of their own judgments (Vogeley et al., 2001). Similarly, preference prediction in the other-trial might involve self-referential social information processing. If preference prediction involved processing of others' information independent of self-reference, preference prediction accuracy should have been correlated with beta oscillations in the other-trials, in addition to discrepancy between the other-trial and the self-trial. However, we observed a significant correlation only in differential beta oscillations between two types of trials, implying that prediction of others' preference might embrace self-referential information processing.

EEG beta oscillations have been implicated in the prediction of one's preference over movie and musical tempo (Popova et al., 2014; Kang et al., 2015). Northoff et al., also suggested that self-referential information processing in the social domain was reflected in neural activity over the regions of mPFC, ACC, temporal pole and superior temporal sulci. Specifically, while medial cortical areas play as a core system, lateral cortical areas are engaged in high-order information processing (Lachat et al., 2012; Kang et al., 2015). Therefore, right temporoparietal beta oscillations may be related to self-referential social information processing of individuals. Moreover, we found that these oscillations could be found in the item phase, not in the target phase, indicating individual performance of preference prediction.

Temporoparietal beta oscillations correlated with individual prediction performance were observed particularly in the right hemisphere. This observation may be related to the role of rTPJ in social information processing reported in many previous studies. For instance, rTPJ has been implicated in understanding of others' mental state (Krall et al., 2015), in shared interpersonal representation between self and others (Decety and Sommerville, 2003), and other social cognitive functions (Decety and Lamm, 2007). In particular, the roles of rTPJ in interpersonal awareness between self and others (Decety and Sommerville, 2003) and in capacity of inferring others' intention, desires, or beliefs (Young et al., 2010) may suggest that the modulation of right temporoparietal beta oscillations shown in this study might reflect the activity of rTPJ to infer preference of others by socially differentiating self-preference.

The timeline of the EEG and behavioral features found in the item phase revealed that right temporoparietal beta oscillations correlated with prediction accuracy appeared earlier than differential alpha ERS between the other-trial and the self-trial, followed by average response time. It may indicate that participants' cognitive system might perform the self-referential information processing before judging others' preference. However, not every aspect of the preference prediction process through thin-slicing could be represented in scalp EEG. Many previous studies reported that frontal areas such as mPFC are primarily involved in the self-referential processing (Frith and Frith, 1999, 2003; Vogeley et al., 2001; Kampe et al., 2003; Platek et al., 2004; Boksem and Smidts, 2015; Jack and Schyns, 2015; Kang et al., 2015), which was not present in the EEG data of this study. Nevertheless, our results may suggest importance of the self-referential information processing for individual performance of others' preference prediction.

It is worthy of remark that the use of a referencing method influenced the EEG analysis results in our study. When we initially used a traditional right-ear mastoid channel as a reference without an additional referencing method, alpha ERS was different between the other-trial and the self-trial over midline and right parietal areas including Pz, P4, and T6 channels. However, when we used the standardized REST method which theoretically provides a constant reference, only the midline parietal area (Pz) exhibited the alpha ERS difference. Also, the correlation between individual beta power and prediction accuracy was observed at the left temporoparietal area (T5) without using REST, but at the right temporoparietal area (T6) after referencing EEG signals by the REST method. Although it requires more in-depth investigations to understand how the change of reference altered spatial aspects of EEG oscillations, our observation may support previous findings that the choice of reference would be crucial to the analysis of scalp EEG data (Yao, 2001; Yao et al., 2005).

Although this study demonstrated the temporal aspect of brain oscillations related to the prediction of others' preference and the putative role of the self-referential information processing in individual prediction performance, other important features of brain activity such as functional connectivity were not investigated, which needs be further pursued in the follow-up studies. In addition, as the behavioral accuracy for prediction preference over food were not reliable, our data analysis was only limited to a single item category. Hence, the same study on different item categories should be carried out to justify the findings in this study.

## Author contributions

All co-authors contributed to the article as follows: JP conducted all aspect of the work, analyzed the data and wrote the manuscript; HK directed literature review and edited the manuscript; JS and JC analyzed the data and edited the manuscript; S-PK oversaw the study, managed every part of research and edited the manuscript.

### Conflict of interest statement

The authors declare that the research was conducted in the absence of any commercial or financial relationships that could be construed as a potential conflict of interest.
